# EXPLORING FACTORS INFLUENCING WALKING ACTIVITY IN PEOPLE WITH DEMENTIA: A SPATIAL ANALYSIS IN METRO VANCOUVER

**DOI:** 10.1093/geroni/igae098.1335

**Published:** 2024-12-31

**Authors:** Mohammadjavad Nouri, Habib Chaudhury

**Affiliations:** Simon Fraser University, Vancouver, British Columbia, Canada; Simon Fraser University, Vancouver, British Columbia, Canada

## Abstract

As the number of people living with dementia (PLWD) increases in Canada, the significance of the neighborhood built environment grows, as most PLWD live at home rather than in long-term care facilities. This study investigates how built environment variables predict the overall outdoor walking activity of PLWD in Metro Vancouver. Twenty-five participants living with dementia from various cities in Metro Vancouver participated. We used exploratory factor analysis and multiple linear regression to analyze the relationship between built environment factors and outdoor walking activity. Our results revealed three significant factors: “Macro environment: accessibility to public transportation and street network,” “Micro environment: Pedestrian-oriented design,” and “General characteristics: Mixed land use and sidewalk suitability,” constructed from 14 built environment variables. These factors significantly influenced the outdoor walking activity of PLWD. The multiple linear regression results indicated that “Macro environment – accessibility to public transportation and street network” had a substantial positive effect (p =.007, β = 0.469), “Micro-environment: Pedestrian-oriented design” showed a moderately positive impact (p =.065, β = 0.305), and “General characteristics: mixed land use and sidewalk suitability” exhibited a significant positive influence (p =.015, β = 0.414) on outdoor walking activity. Our findings provide valuable insights for policymakers, urban planners, designers, landscape designers, and transportation planners, emphasizing the crucial collaboration needed to develop dementia-friendly neighborhood plans that cater to the diverse needs of individuals living with dementia, ensuring safe and accessible environments.

